# Numerical Investigations of Urban Pollutant Dispersion and Building Intake Fraction with Various 3D Building Configurations and Tree Plantings

**DOI:** 10.3390/ijerph19063524

**Published:** 2022-03-16

**Authors:** Qingman Li, Jie Liang, Qun Wang, Yuntong Chen, Hongyu Yang, Hong Ling, Zhiwen Luo, Jian Hang

**Affiliations:** 1 Southern Marine Science and Engineering Guangdong Laboratory (Zhuhai), School of Atmospheric Sciences, Sun Yat-sen University, Zhuhai 519082, China; liqm23@mail2.sysu.edu.cn (Q.L.); liangj58@mail2.sysu.edu.cn (J.L.); chenyt226@mail2.sysu.edu.cn (Y.C.); yanghy46@mail2.sysu.edu.cn (H.Y.); hangj3@mail.sysu.edu.cn (J.H.); 2 Key Laboratory of Tropical Atmosphere-Ocean System, Ministry of Education, Sun Yat-sen University, Zhuhai 519000, China; 3 Department of Mechanical Engineering, The University of Hong Kong, Pokfulam Road, Hong Kong SAR, China; qunwang@connect.hku.hk; 4 School of Construction Management and Engineering, University of Reading, Whiteknights, Reading RG6 6AH, UK; z.luo@reading.ac.uk

**Keywords:** CFD simulation, ventilation, pollutant dispersion, open space, urban tree planting, personal intake fraction

## Abstract

Rapid urbanisation and rising vehicular emissions aggravate urban air pollution. Outdoor pollutants could diffuse indoors through infiltration or ventilation, leading to residents’ exposure. This study performed CFD simulations with a standard *k-ε* model to investigate the impacts of building configurations and tree planting on airflows, pollutant (CO) dispersion, and personal exposure in 3D urban micro-environments (aspect ratio = *H/W* = 30 m, building packing density *λ_p_* = *λ_f_* = 0.25) under neutral atmospheric conditions. The numerical models are well validated by wind tunnel data. The impacts of open space, central high-rise building and tree planting (leaf area density *LAD*= 1 m^2^/m^3^) with four approaching wind directions (parallel 0° and non-parallel 15°, 30°, 45°) are explored. Building intake fraction <*P_IF*> is adopted for exposure assessment. The change rates of <*P_IF*> demonstrate the impacts of different urban layouts on the traffic exhaust exposure on residents. The results show that open space increases the spatially-averaged velocity ratio (*VR*) for the whole area by 0.40–2.27%. Central high-rise building (2*H*) can increase wind speed by 4.73–23.36% and decrease the CO concentration by 4.39–23.00%. Central open space and high-rise building decrease <*P_IF*> under all four wind directions, by 6.56–16.08% and 9.59–24.70%, respectively. Tree planting reduces wind speed in all cases, raising <*P_IF*> by 14.89–50.19%. This work could provide helpful scientific references for public health and sustainable urban planning.

## 1. Introduction

Rapid urbanisation has aggravated urban environmental problems over the past several decades. The rapidly increasing vehicular emissions in street networks deteriorate urban air quality and have become one of the main pollutant sources in modern cities [1,2,3]. Urban air pollutant exposure has induced rising risks of respiratory and cardiovascular diseases, or even premature mortality [4,5]. People spend more than 90% of their lifetime indoors, on average. Moreover, outdoor air pollutants can diffuse into the indoor environment by infiltration or ventilation via windows, vents, and so on. The indoor pollutant exposure is closely influenced by the outdoor air quality, especially for buildings with natural ventilation. Therefore, near-road residents usually suffer from much higher air pollutant exposure than those in other regions [4,6,7,8]. Special attention is required to develop sustainable urban designs to improve urban ventilation and reduce urban residents’ exposure [9,10].

The urban canopy layer (UCL) represents the atmospheric layer from the ground to the building rooftops, where most urban residents live. To mitigate the pollutant exposure of residents in the UCL, improving the ventilation and pollutant dilution capacity is one of the major solutions [9,10]. Recently, field observations, computational fluid dynamic (CFD) simulations, and laboratory-scale physical modelling (wind tunnel or water channel experiments) have been widely employed to investigate the ventilation and pollutant dispersion at the street scale (~100 m) or neighbourhood scale (~1 km) [11,12,13,14,15,16,17,18]. Field observation can directly monitor the critical characteristics of air flow and dispersion in real cities, but is restricted by low spatial resolution, uncontrollable boundary conditions, and complicated building configurations [15]. Although laboratory-scale physical modelling techniques can control boundary conditions and building configurations well, and have been widely used to validate numerical models, they have to meet the similarity criteria requirements and the costs are relatively high [13,15,17,18]. Numerical modelling with a high temporal–spatial resolution turns out to be a more efficient and relatively low-cost tool to study the flow features and dispersion characteristics, but sometimes it has challenges in attaining satisfactory validation by experimental data [19,20,21,22,23,24,25]. In this work, CFD modelling is applied to simulate the flow field and pollutant dispersion at the street scale.

Key urban morphological parameters include street aspect ratio (*AR* = *H/W*, where *H* is the building height, *W* is the street width) [26,27], tree planting [28,29,30], the direction of approaching flow [31,32], building packing densities [33,34], building height variation [35,36], special building designs including open space [37,38,39] and high-rise building [40,41]. Previous studies have investigated their impacts on the urban ventilation and pollutant dispersion. Nevertheless, while most studies focused on the airflow and pollutant dispersion in the street canyon, the integrated impacts of different urban layouts on residents’ exposure in three-dimensional (3D) urban models are still rare. Therefore, this study aims at evaluating the synthetic impacts of these urban parameters (urban open space, tree planting and central high-rise building in this work) on ventilation, pollutant dispersion and related human exposure. The work provides a scientific reference and effective methodologies for sustainable urban design and public health.

## 2. Methodology

### 2.1. Definition of Crucial Parameters

#### 2.1.1. Velocity Ratio (*VR*)

Velocity ratio (*VR*) is used throughout the work to normalise and quantify the wind environment experienced by pedestrians [11], defined by Equation (1):

(1) $$VR=V_{p}/V_{\delta }$$

where *V_p_* is the wind velocity at the pedestrian level (*z* = 2 m) and *V*_δ_ is the wind velocity at the top of the boundary layer. Here, *V*_δ_ = 4.34 m/s at *z* = 300 m [39].

#### 2.1.2. Building Intake Fraction <*P_IF*>

The concept of the building intake fraction <*P_IF*>, derived from the personal intake fraction *P*_*IF*, is employed to evaluate the impacts of urban layouts on the residents’ pollutant exposure. *P*_*IF* represents the total pollutant inhalation per person, which is widely adopted to quantify the indoor and street-scale (~100 m) vehicular pollutant exposure [42,43,44].

The intake fraction (*IF*) for a certain population is defined in Equation (2) [45,46,47,48]:

(2) $$IF=\overset{N}{\underset{i}{\sum }}\overset{M}{\underset{j}{\sum }}P_{i}\times Br_{i,j}\times \Delta t_{i,j}\times Ce_{j}/m$$

where *N* stands for the total number of population age groups considered in this research; *M* is the total number of micro-environment types; *P_i_* is the number of the population in the age group *i*; *Br_i,j_* (m^3^/s) is the volume-mean breathing rate for individuals of the age group *i* in the micro-environment group *j*; *∆t_i,j_* is the time that group *i* stays in the micro-environment *j*; *Ce_j_* (kg/m^3^) is the time-averaged concentration *C* of the certain vehicular pollutant in the micro-environment *j*; and *m* (kg) is the total emission of the vehicular pollutant over the research period.

The population in the research is divided into three groups (*N* = 3) according to Luo et al. [46]. The composition of the target population is: children (<18, *i* = 1), adults (18–60, *i* = 2) and elders (>60, *i* = 3). Chau et al. [49] considered four types of micro-environment (*M* = 4) in their work, including indoor at home (*j* = 1), other indoor locations (*j* = 2), near vehicles (*j* = 3) and other outdoor locations (*j* = 4). To simplify the computation, only one micro-environment (*j* = 1, indoor at home) is considered in this study, and the buildings are assumed to be the residential type with natural ventilation. As Table A1 in the supplement shows, the percentage of children, adults and elders are 21.2%, 63.3% and 15.5%, respectively, in this paper. The breathing rate *Br* and time percentage spent indoors at home for different age groups is 12.5 and 61.70% (children), 13.8 and 59.50% (adults) and 13.1 and 71.60% (elders), respectively [46,49,50].

*IF* has been used to express the source-to-intake relationship for vehicular pollutants in realistic street canyons [48]. Since *IF* would change linearly with the variation of the population, it has been optimised by defining personal intake fraction *P_IF* in Equation (3) [26,42]. *P_IF* is independent of population size and density, and it represents the average *IF* for each person.

(3) $$P\_IF=IF/\sum_{j}^{M}P_{i}$$

To estimate the influence of urban layouts on personal exposure, *P_IF* is employed in this work to quantitatively evaluate the pollutant inhalation of residents.

Previous researchers [6,7] found that the ratio of indoor and outdoor pollutant concentration (I/O) was approximately 1 for buildings with natural ventilation. Therefore, to reduce the number of grids and computational resources, the inner space of buildings is not considered in simulations. The pollutant concentrations on building surfaces are adopted as the indoor concentrations in this work, and the vehicle emission is assumed as the only source of the indoor environment [26,43,45]. Building intake fraction <*P_IF>* is the spatial mean of *P_IF* at all building surfaces. Throughout this work, <*P_IF>* is used to present the spatially-averaged personal intake exposure for the whole urban area. The change rate of *<P_IF>* could represent the varied exposure risks for the indoor residents due to the impacts of different urban layouts.

### 2.2. Set-Up for Numerical Modelling

CFD simulation has been widely used for urban micro-climate research in recent decades [51,52,53,54,55]. Compared with Reynolds-averaged Navier–Stokes (RANS) approaches, large eddy simulations (LES) are more accurate in simulating and predicting turbulence [54,56,57,58,59,60]. However, LES models need enormous computational resources. Thus, RANS models are still widely applied for turbulence simulation [61,62,63,64,65]. Among the RANS models, the standard *k-ε* model has remarkable performance in predicting urban airflows and pollutant dispersion [27,33,44,66,67,68]. In this paper, the Ansys FLUENT 15.0 with standard *k-ε* model is applied for airflow simulations under isothermal conditions.

#### 2.2.1. CFD Model Description

The governing equations of mass, momentum, turbulent kinetic energy (*k*) and its dissipation rate (*ε*) of the employed CFD model are shown in Equations (4)–(7), as follows: the mass conservation equation:

(4) $$\frac{\partial \bar{u_{i}}}{\partial x_{i}}=0$$

the momentum equation:

(5) $$\bar{u_{j}}\frac{\partial \bar{u_{i}}}{\partial x_{i}}=-\frac{1}{\rho }\frac{\partial \bar{p}}{\partial x_{i}}+\frac{\partial }{\partial x_{j}}\left(v\frac{\partial \bar{u_{i}}}{\partial x_{j}}-\bar{{u_{i}}^{\prime \prime }{u_{j}}^{\prime \prime }}\right)$$

the transport equations of turbulent kinetic energy (*k*) and its dissipation rate (*ε*):

(6) $$\bar{u_{i}}\frac{\partial k}{\partial x_{{}_{i}}}=\frac{\partial }{\partial x_{{}_{i}}}\left[\left(v+\frac{v_{{}_{t}}}{\sigma_{{}_{k}}}\right)\frac{\partial k}{\partial x_{{}_{i}}}\right]+\frac{1}{\rho }P_{{}_{k}}-\varepsilon$$

(7) $$\bar{u_{{}_{i}}}\frac{\partial \varepsilon }{\partial x_{{}_{i}}}=\frac{\partial }{\partial x_{{}_{i}}}\left[\left(v+\frac{v_{{}_{t}}}{\sigma_{{}_{\varepsilon }}}\right)\frac{\partial \varepsilon }{\partial x_{{}_{i}}}\right]+\frac{1}{\rho }C_{{}_{\varepsilon 1}}\frac{\varepsilon }{k}\left(P_{{}_{k}}+C_{{}_{\varepsilon 3}}G_{{}_{b}}\right)-C_{{}_{\varepsilon 2}}\frac{\varepsilon^{2}}{k}$$

where ${\bar{u}}_{j}$ stands for time-averaged velocity components (${\bar{u}}_{j}=\bar{u},\bar{v},\bar{w}$ as *j* = 1, 2, 3); v is the kinematic viscosity; and $v_{{}_{t}}$ is the kinetic eddy viscosity ($v_{{}_{t}}=C_{\mu }\frac{k^{2}}{\varepsilon }$). The constant $C_{\mu }$ is 0.09. $-\bar{{u^{\prime \prime }}_{i}{u^{\prime \prime }}_{j}}=v_{t}\left(\frac{\partial \bar{u_{i}}}{\partial x_{i}}+\frac{\partial \bar{u_{j}}}{\partial x_{j}}\right)-\frac{2}{3}k\delta_{ij}$ is the Reynolds stress tensor. $\delta_{ij}$ is the Kroneker delta. $\delta_{ij}$ = 1 when i=j and $\delta_{ij}$ = 0 otherwise. $P_{k}=v_{t}\times \frac{\partial \bar{u_{i}}}{\partial x_{j}}\left(\frac{\partial \bar{u_{i}}}{\partial x_{j}}+\frac{\partial \bar{u_{j}}}{\partial x_{i}}\right)$ is the turbulence production term.

The SIMPLE scheme is applied for coupling pressure and velocity. The under-relaxation factors for pressure term, momentum term, *k* and *ε* terms are 0.3, 0.7, 0.5 and 0.5, respectively. When all the absolute residuals are smaller than 10^−6^, the iteration is converged.

#### 2.2.2. Model Set-Up and Boundary Conditions

**Figure 1:**
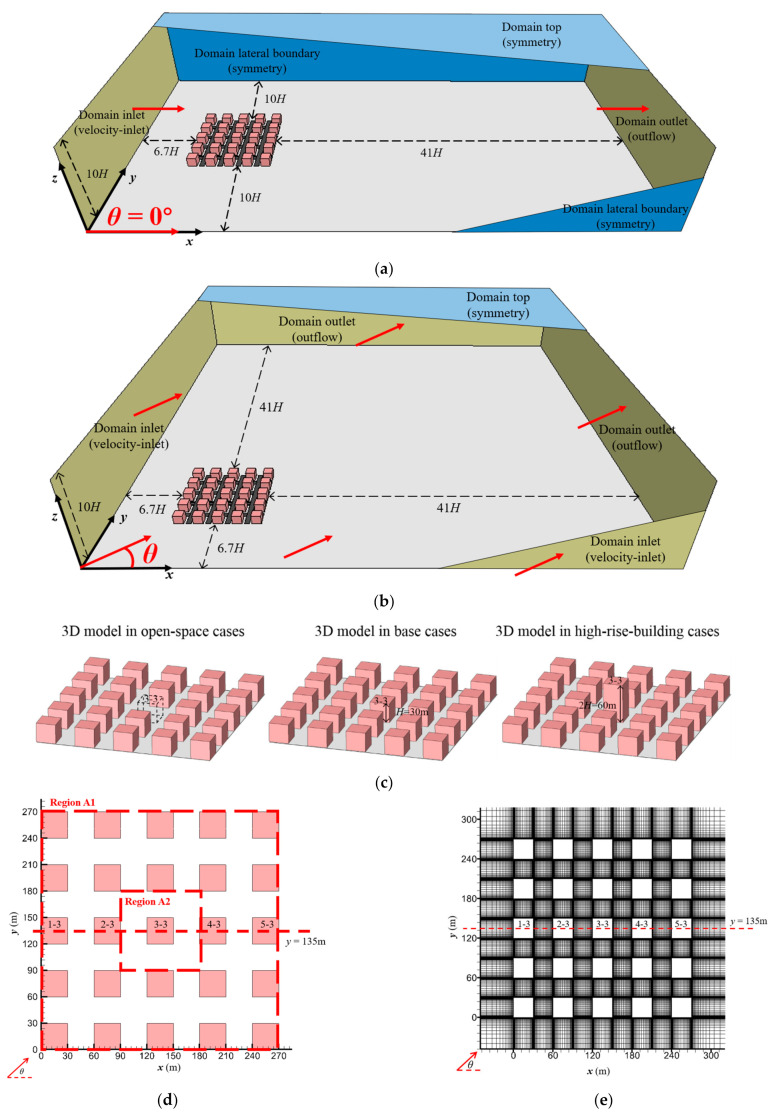

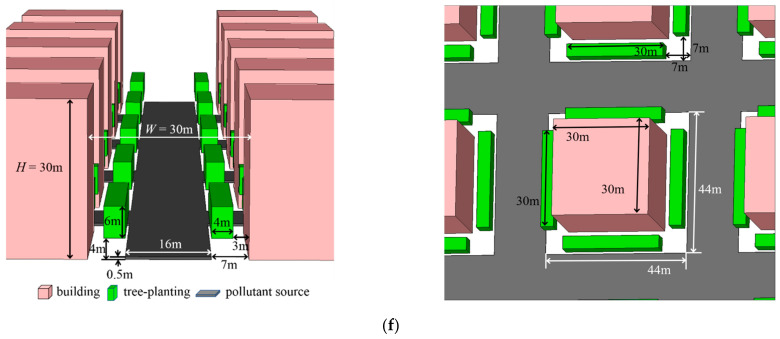
Computational domain of (**a**) Case [Base, 0°] and (**b**) Case [Base, *θ*] (*θ* = 15°, 30°, 45°). (**c**) 3D model description of open-space cases, base cases and high-rise-building cases. (**d**) Model description and (**e**) grid arrangements from top view in base cases. (**f**) Setups of building, tree planting and pollutant source.

The 3D idealised full-scale UCL model with neutral atmosphere conditions is adopted in this study. The whole UCL model has a 5 × 5 building matrix composed of 25 cubic models (*H* = *B* = *W* = 30 m) with moderate packing density (aspect ratio *H/W* = 1, building packing density *λ_p_* = *λ_f_* = 0.25). The designed UCL model is an idealised typical urban residential area in miniature, especially relating to the communities in small and medium-sized towns, or the communities in the old town of modern cities. To evaluate the impacts of approaching winds with different directions, *θ* (the included angle of the approaching wind and axis *x*) is set as 0°, 15°, 30° and 45° for every scenario. The setup of the simulation domain is shown in Figure 1a,b.

Figure 1a depicts the simulation area of the cases with parallel approaching wind (*θ* = 0°), with the geometry of 1700 m (*x*) × 870 m (*y*) × 300 m (*z*). The distances from the UCL model to the domain inlet, outlet and lateral boundaries are 6.7*H*, 41*H* and 10*H*, respectively. The symmetry boundary condition is adopted at the domain top and the lateral boundaries, while the domain outlet takes the zero normal gradient boundary condition [39,69,70].

The domain geometry of the cases with non-parallel approaching wind (*θ* = 15°, 30° and 45°) is 1700 m (*x*) × 1700 m (*y*) × 300 m (*z*) (Figure 1b). In this condition, the distances from the UCL model to the domain inlets and outlets are 6.7*H* and 41*H*, respectively. The symmetry boundary condition is only adopted at the domain top.

The boundary condition for the domain inlet is provided by Equations (8)–(10) [27,67,71,72]:

(8) $$U_{in}\left(z\right)=U_{ref}\times {\left(z/H\right)}^{0.16}$$

(9) $$k_{in}\left(z\right)=u^{*}{}^{2}/\sqrt{C_{\mu }}$$

(10) $$\varepsilon_{in}\left(z\right)=C_{\mu }^{\frac{3}{4}}k_{in}^{\frac{3}{2}}/\left(\kappa_{v}z\right)$$

where at the building height (*H* = 30 m), the reference velocity $U_{ref}$ is 3 m/s. The friction velocity $u^{*}$ is 0.24 m/s. The von Kármán constant $\kappa_{v}$ is 0.41. The empirical constant $C_{\mu }$ is 0.09.

Six building configurations are considered in the current study (Table 1). The UCL model of base cases is a building matrix with 5-row and 5-column blocks (Figure 1c). For the study of the open-space effect on city ventilation and human exposure, we remove the central building (Building 3-3) to obtain the open space (Location 3-3). For high-rise building scenarios, we double the height of Building 3-3. Each type of building configuration is combined with tree-planting and tree-free types. According to the settings above, the cases are named “Case [urban layout, wind direction]”, as shown in Table 1.

For the comprehensive data analysis, we present the results both in the entire UCL Region A1 (5 × 5 building matrix) and in the Region A2 (the central area of Region A1, including Building 3-3/Location 3-3 and the surroundings), as shown in Figure 1d. For all tested cases, the minimum size of the hexahedral cells near wall surfaces is 0.2 m. The total number of grids ranges from approximately 5 million and 15 million for the tree-planting and tree-free cases, respectively. Figure 1e illustrates the grid arrangement from the top view and side view. This grid arrangement is sufficient to ensure the requirement recommended by CFD guidelines [69,70].

#### 2.2.3. Description of Pollutant Dispersion Modelling

Scientists have found that traffic emissions have critical negative impacts on respiratory and cardiovascular function [73,74]. The situation in Asian cities might be more severe due to the very high population density and more residents living in close proximity to road traffic compared with those in European cities. To model the dispersion of traffic-related pollutants in the street canyon, reactive gaseous as primary particles [75], inert gas like CO [76], as well as reactive gaseous pollutants such as NO_x_ and VOCs [77] are adopted in the simulation for investigating the dispersion of traffic-related air pollutants. Unlike various studies focusing on the reactive compounds from traffic such as NO_x_, VOCs and particles, we adopted monoxide (CO) as an indicator of the traffic emissions. Although NO_x_, VOCs and particles have more significant health impacts than CO, these compounds are more or less chemically or photo-chemically reactive, which means they could not be used as a tracer for the variation of the traffic emissions. To numerically investigate the impacts of various urban layouts on the physical dispersion of the traffic-related pollutants and related human exposure, a stable indicator is needed. As one of the main inert pollutants, CO has been widely used as a tracer of traffic emissions [42,46,48]. This study mainly focuses on the dynamic dispersion of traffic pollutants influenced by different urban layouts. Deposition and chemical reactions are not considered. The CO emission source is settled from *z* = 0 m to 0.5 m, with a width of 16 m and is 7 m away from the kerbside building (marked with dark grey colour in Figure 1f).

The governing equation of time-averaged CO concentration *C* (kg/m^3^) is applied as Equation (11):

(11) $$\bar{u_{j}}\frac{\partial C}{\partial x_{j}}-\frac{\partial }{\partial x_{j}}\left[\left(D_{m}+D_{t}\right)\frac{\partial C}{\partial x_{j}}\right]=S$$

where $\bar{u_{j}}$ is the time-averaged velocity component in the direction of *j*. $D_{m}$ and $D_{t}$ are molecular diffusivity and the turbulent diffusivity of the pollutant. $D_{t}=\nu_{t}/S_{Ct}$, while $\nu_{t}$ is the kinematic eddy viscosity, and $S_{Ct}$ is the turbulent Schmidt number. It is a parameter describing an important property of the flow defined as the ratio of the eddy diffusivity of momentum to the eddy diffusivity of mass. Di Bernardino et al. [78] found that $S_{Ct}$ increased with the height above the canopy, with the maxima of about 0.6 in their water-channel experiments and simulations. In our preliminary work, we found that modelling with $S_{Ct}$ = 0.7 had the best performance compared with the wind tunnel experiment results from Gromke and Blocken [63]. Thus, $S_{Ct}$ = 0.7 is used throughout this work. The CO emission rate *S* is set as 1.25 × 10^−6^ kg/m^3^/s, derived from a field observation campaign in a real street of Hong Kong [79]. Such an emission source setting has been adopted in CFD simulations for many studies of urban pollutant dispersion [10,27,42,80].

#### 2.2.4. Description of the Vegetation Modelling

Tree planting is simulated as a series of cubic blocks on both sides of the streets over the whole urban area, except the surroundings of Building 3-3 (Figure 1f). Since the size of the tree trunk is much smaller than that of the crown, the impact of the trunk on the airflow is assumed to be negligible, therefore only the crowns are simulated in the tree-planting cases. According to Yang et al. [80], the *y-density* is set to 1, which means that the tree crown is continuous in the *y*-direction. As shown in Figure 1f, the scale of the crown cubic is designed as 4 m × 6 m × 30 m. The distance between the crown bottom and the ground surface is 4 m, and that between the crown and the adjacent building wall is 3 m.

Differing from solid obstacles such as buildings, the airflow can pass through the tree crown from spaces in between the branches and leaves. Previous studies found that vegetation models with the porous medium for airflow and pollutant dispersion were more consistent with the wind tunnel experimental results than those with a non-porous medium [81,82,83]. Accordingly, we adopt the porous fluid zones to simulate tree planting, and the governing equations are listed in Equations (12)–(14).

(12) $$S_{\bar{u_{i}}}=-\rho C_{d}LAD\bar{u_{i}}U$$

(13) $$S_{k}=\rho C_{d}LAD\left(\beta_{p}U{\bar{u_{i}}}^{3}-\beta_{d}Uk\right)$$

(14) $$S_{\varepsilon }=\rho C_{d}LAD\frac{\varepsilon }{k}\left(C_{\varepsilon 4}\beta_{p}U^{3}-C_{\varepsilon 5}\beta_{d}Uk\right)$$

where $S_{\bar{u_{i}}}$, $S_{k}$, $S_{\varepsilon }$ are the additional source and sink terms of momentum, turbulent kinetic energy and turbulent dissipation rate for trees, respectively. ρ (kg/m^3^) is the air density.$\;C_{d}$ is the leaf drag coefficient, ranging from 0.1 to 0.3, which is related to the tree species. In this paper, we adopt the commonly used empirical value of $C_{d}$ = 0.2 to avoid species particularity [81]. *LAD* (m^2^/m^3^) is the leaf area density, which represents the one-side leaf area per unit volume of the crown [80,84]. It is related to tree species and crown-height variations, and ranges between 0.5 and 2.0 m^2^/m^3^ for deciduous trees. To simplify the model, we supposed that the trees in the simulation domain were all deciduous trees with a homogeneous crown height, and thus set the *LAD* value to 1 m^2^/m^3^ for the simulation. $\bar{u_{i}}$ is the time-averaged velocity component on direction *i*, and ***U*** is the magnitude of the velocity. $\beta_{p}$ is the portion of turbulent kinetic energy converted from mean kinetic energy under the influence of drag, and $\beta_{d}$ is the dimensionless coefficient of the Kolmogorov cascade. We adopt $\beta_{p}$ as 1.0, and $\beta_{d}$ as 5.1, according to [81,82], respectively. Both $C_{\varepsilon 4}$ and $C_{\varepsilon 5}$ are empirical constants of 0.9.

#### 2.2.5. Validations for Flow, Dispersion and Vegetation Modelling

The direct validation for the CFD model of real urban areas is difficult due to the very limited field observation data. The uncontrollable boundary condition is another challenge for repetitive experiments [85]. However, the wind tunnel experiment is a credible solution for model validation if the Reynolds number (*Re*) independence is satisfied (*Re* >> 11,000) [86,87,88]. We have implemented a series of comprehensive validations for the flow, the dispersion and the vegetation model applied throughout this work, based on the published wind tunnel experiment datasets [86,89,90]. Similar validation methods have been employed and proven valid in the literature [43,80,88].

- *Flow validation by wind tunnel tests of cubic arrays*

**Figure A1:**
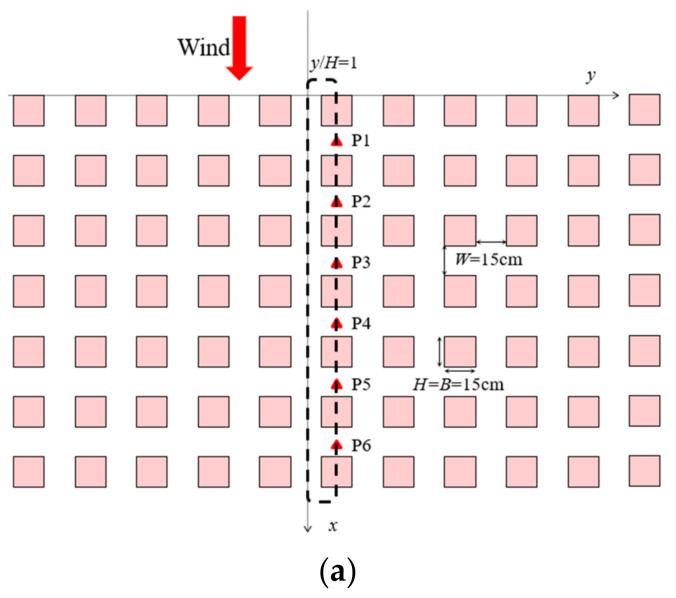

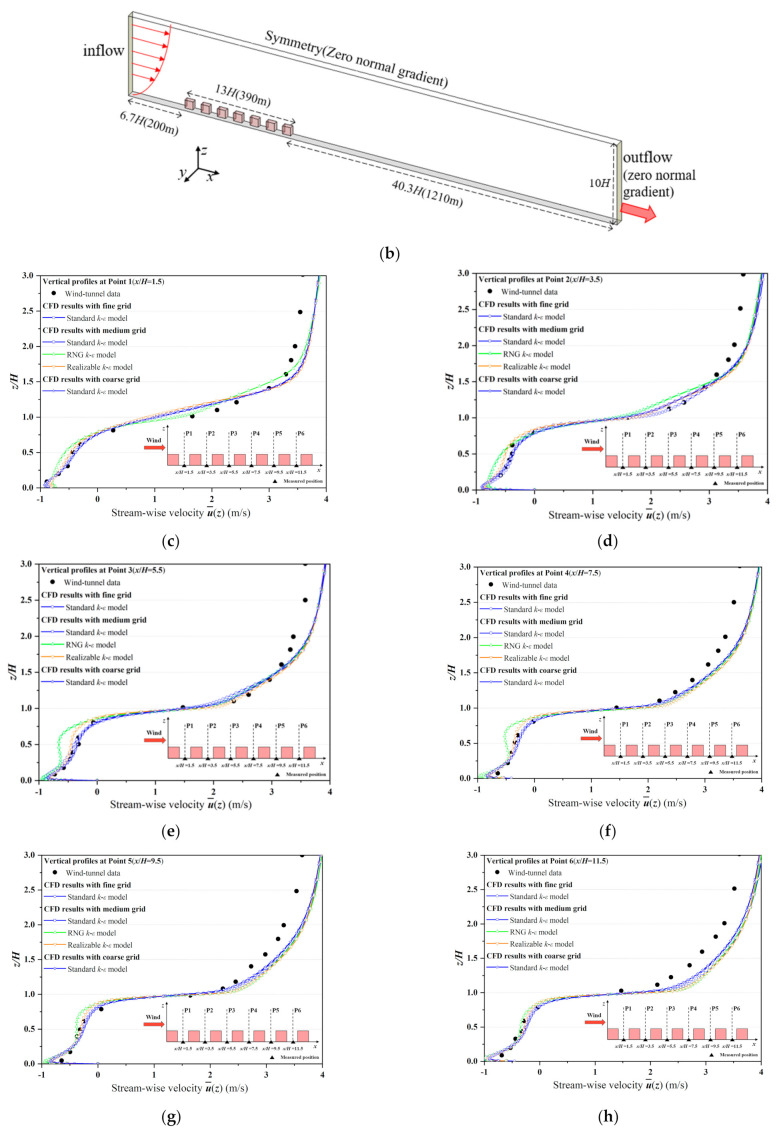

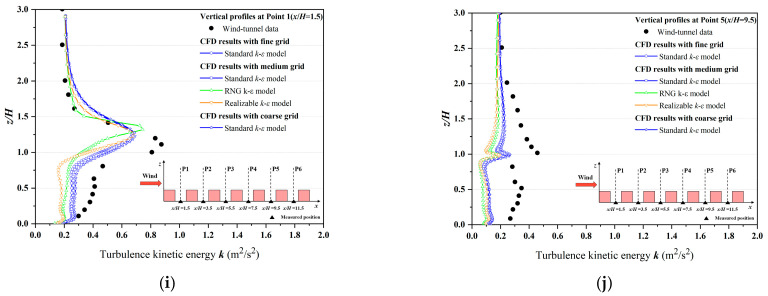
Flow validation by wind tunnel data. (**a**) Geometry of the UCL model in wind tunnel dataset. (**b**) Setting of the computational domain and boundary conditions. (**c**–**h**) Vertical profiles of monitored and modelled stream-wise velocity ($\bar{u}$) at Point P1–P6. (**i**,**j**) Vertical profiles of monitored and modelled turbulence kinetic energy (*k*) at Point P1 and P5.

Figure A1 presents the results of the flow validation. The UCL model (moderate building density) with a 7 × 11 building matrix was used in the employed wind tunnel dataset [86]. The size of each building model is *H* = *B* = *W* = 15 cm. The measuring points for the vertical profiles of the stream-wise velocity ($\bar{u}$) and turbulence kinetic energy (*k*) are set in the centre of each street, named P*i* (*i* = 1–6) (Figure A1a,b). As we described in Section 2.2.2, similar model configurations are set for the case studies: at full scale with a scale ratio of 200:1 (*H* = *B* = *W* = 30 m) to the wind tunnel scale. All settings are similar, except the length from the urban boundary to the domain outlet. Referring to the reference velocity (*U_ref_* = 3 m/s) and the model geometry (*H* = 0.15 m or 30 m), *Re* is approximately 3 × 10^4^ and 6 × 10^6^ at the wind tunnel scale and full scale, satisfying the requirements for Reynolds number independence.

Figure A1c–j tests the grid independence (with a minimum grid size of 0.4 m, 0.2 m and 0.1 m) and the performance of different turbulence models (standard *k-ε*, RNG *k-ε* and realisable *k-ε* models) with standard wall function. The results illustrate that differences generated by the mesh setting are negligible. Thus, the moderate grid size (0.2 m for minima) is applied for all cases to save computational resources. Furthermore, the depicted vertical profiles of $\bar{u}$ and *k* verify that modelling using the standard *k-ε* model has better agreement with the wind tunnel data than those using the RNG *k-ε* and realisable *k-ε* models. Important statistics are summarised in Table A2, including the normalised mean square error (NMSE), fractional bias (FB) and correlation coefficient (R). The results denote that the standard *k-ε* model is employed in the study throughout this work.

- *Pollutant dispersion validation by wind tunnel tests without tree models*

**Figure A2:**
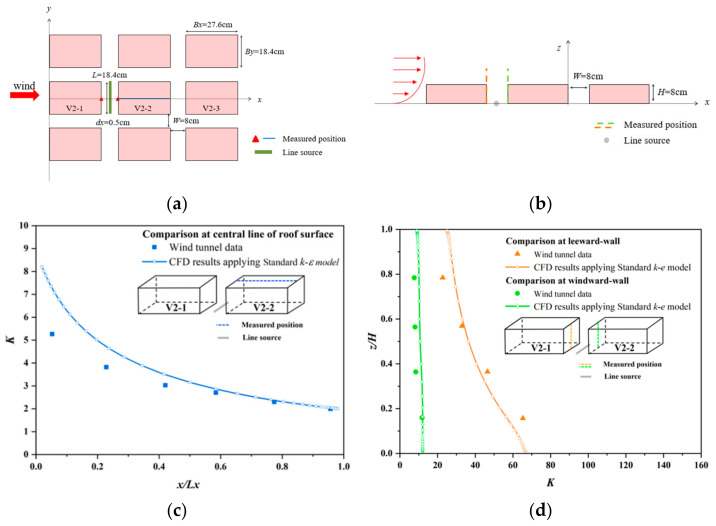
Validation for pollutant dispersion. (**a**) Configurations of the wind tunnel experiment with top view. (**b**) Configurations of the wind tunnel experiment with lateral view. (**c**) Vertical profiles of normalized inert gas concentration *K* at the roof top of the model. (**d**) Vertical profiles of *K* at the leeward and windward wall.

Wind tunnel experiment data of inert gas dispersion [89] is employed in our work for the validation of pollutant dispersion. The configurations of the experiment are illustrated in Figure A2a,b. The UCL model consists of a 3 × 3 model matrix, with each prism size being *B_x_* × *B_y_* × *H* = 27.6 cm × 18.4 cm × 8 cm. Inert gas C_2_H_6_ is used as the tracer, emitting from the line source (*L* = 18.8 cm, *d_x_* = 0.5 cm) settled in the UCL model area. Similar model settings at full scale (*B_x_* × *B_y_* × *H* = 138 m × 92 m × 40 m) are configured for the simulation to validate the dispersion. Fitting vertical profiles of monitored $\bar{u}$, *k* and *ε* in the wind tunnel experiments [89] are set for the domain inlet. The standard *k-ε* model and standard wall function are adopted in the simulation. Since the tracer gas concentration provided by the wind tunnel experiment is in a non-dimension form, the normalised concentration *K* [89] is derived referring to Equation (15), in convenience for comparing the experimental data and simulation results.

*K* = *C*·*H*·*U_ref_*/*E*·*d_x_* (15)

where *C* is the inert gas concentration, and the emission rate *E* is 0.01 m/s. Important statistics are summarised in Table A3, including the NMSE, FB and R. The good agreement between the results of the wind tunnel experiment and the CFD simulation confirm that the selected turbulence model and wall function is appropriate for evaluating the pollutant dispersion in our work.

- *Pollutant dispersion validation by wind tunnel tests with tree models*

**Figure A3:**
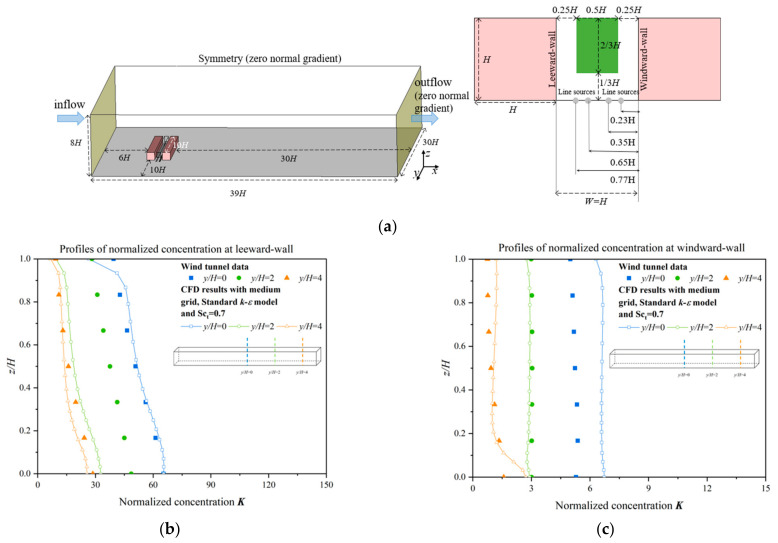
Validation for the vegetation modelling. (**a**) Configurations of the wind tunnel experiment with vegetation model. (**b**) Vertical profiles of *K* at the leeward wall. (**c**) Vertical profiles of K at the windward wall.

The validation for vegetation modelling in this paper is performed on the basis of the wind tunnel experiment conducted by Gromke and Ruck [90]. The configurations and boundary conditions are set as in Figure A3a. The 2D street canyon is constructed by two parallel building models, with the same sizes for both in the wind tunnel experiment and the CFD simulation (*L × W × H* = 1.2 m × 0.12 m × 0.12 m). The standard *k-ε* model and standard wall function coupled with the porous crown model (details in Section 2.2.4) are adopted in the simulation. The vertical profiles of $\bar{u}$, *k* and *ε* for the domain inlet are provided by Gromke and Ruck [90]. *U_ref_* = 4.7 m/s is applied; thus, the reference *Re* is 38,630 >> 11,000. The normalised concentration *K* of the inert gas (SF_6_) is used to compare the results of the wind tunnel experiment and the simulation. Vertical profiles of *K* are presented in Figure A3b,c. Important statistics are summarised in Table A4, including the NMSE, FB and R. In general, the results satisfy the recommended criteria [91,92], except that of *y/H* = 2 at the leeward wall. Nevertheless, as we focus on the pollutant dispersion in the 2D street canyon, the results of *y/H* = 0 (central region of the canyon with fully developed turbulence) are more representative of the pollutant distribution features. Good agreements of these statistics at *y/H* = 0 also confirm the modelling accuracy and reliability of the simulations. These results verify that the porous crown model with the standard *k-ε* model and standard wall function has good performance, and is suitable for studying the tree-planting effects in this work.

## 3. Results

### 3.1. Impacts of Building Configurations and Tree Planting on Flow Pattern

#### 3.1.1. Impact of Open Space and High-Rise Building on Airflow

**Figure 2:**
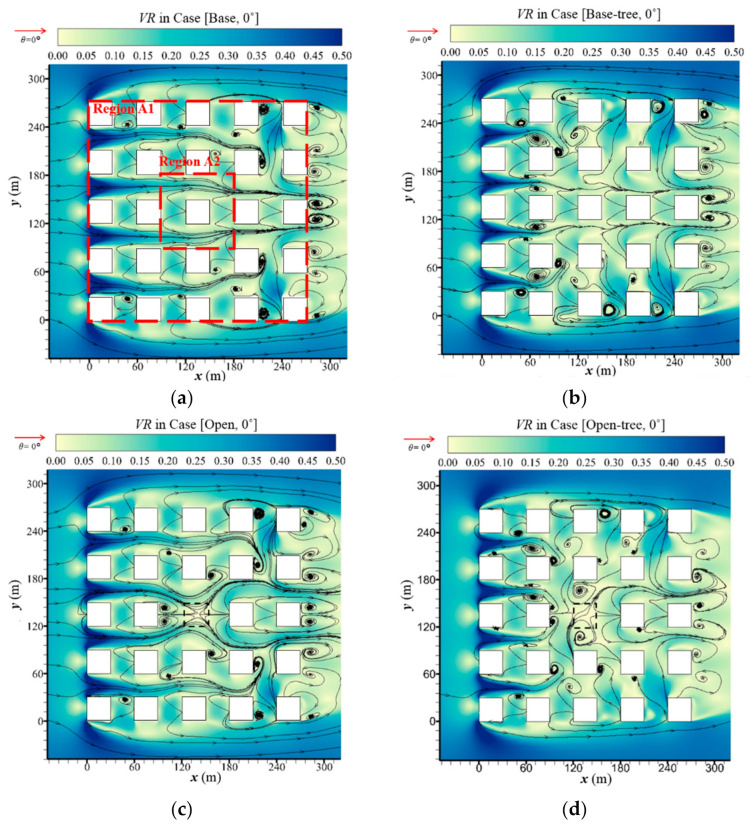

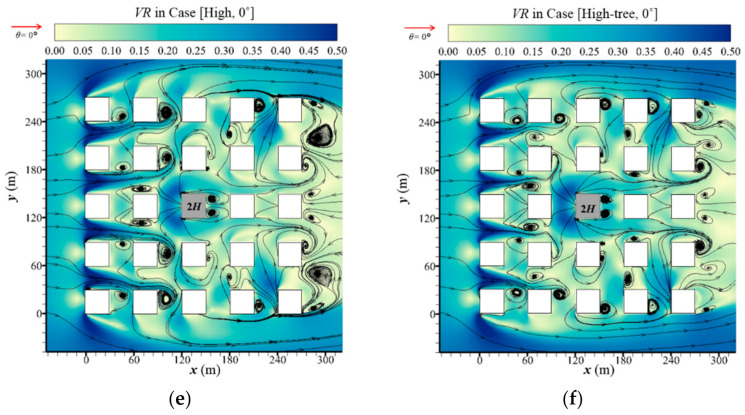
Streamline and velocity ratio (*VR*) at *z* = 2m in (**a**) Case [Base, 0°], (**b**) Case [Base-tree, 0°], (**c**) Case [Open, 0°], (**d**) Case [Open-tree, 0°], (**e**) Case [High, 0°] and (**f**) Case [High-tree, 0°].

Figure 2a,c,e presents the streamlines and velocity ratio (*VR*) at the pedestrian level (*z* = 2 m) of tree-free cases under the parallel approaching wind, named Case [Base, 0°], Case [Open, 0°] and Case [High, 0°]. It shows the impacts of open space and high-rise buildings on the ambient airflows. Both building configurations are found to apparently change the structure of the vortex and airflow, especially within Region A2. *VR* in the downstream regions of Case [Open, 0°] is slightly strengthened compared with Case [Base, 0°]. However, the mean wind speed of Case [Base, 0°] and Case [Open, 0°] in the whole region are at the same level. New vortices are generated around Region A2 with the addition of the open area. Comparing to the base cases, *VR* value increases significant0ly with the addition of high-rise building (Case [High, 0°]), especially in Region A2. The flow structure is changed in the whole urban area, and new vortices are found near the leeward wall or lateral wall of most buildings.

**Figure 3:**
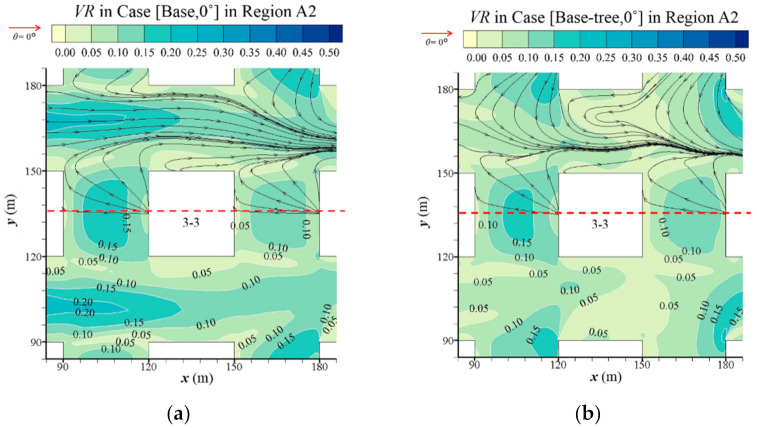

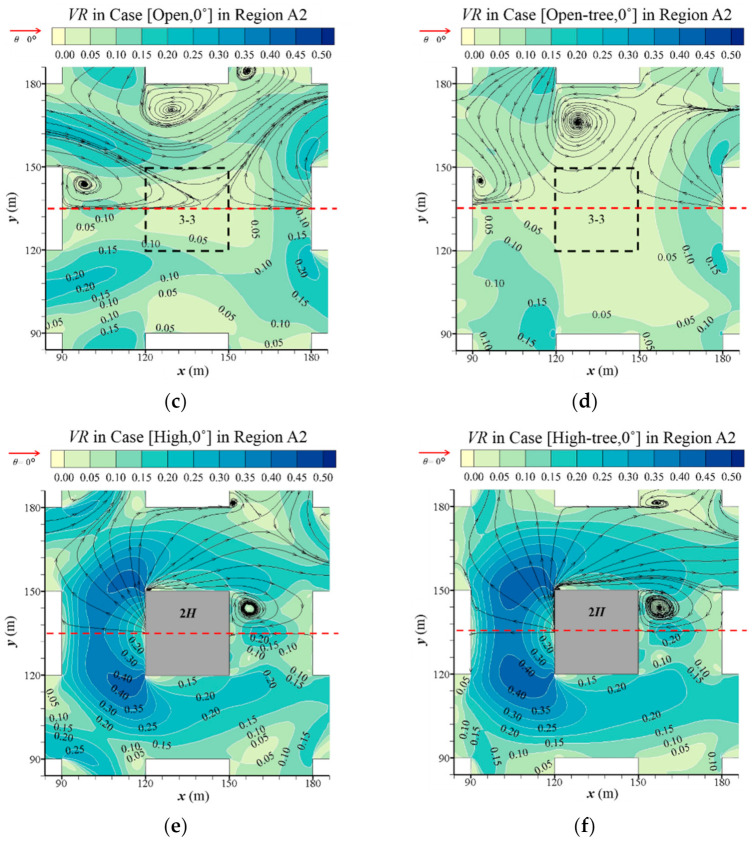
Streamline and velocity ratio (*VR*) at *z* = 2m in Region A2 in (**a**) Case [Base, 0°], (**b**) Case [Base-tree, 0°], (**c**) Case [Open, 0°], (**d**) Case [Open-tree, 0°], (**e**) Case [High, 0°] and (**f**) Case [High-tree, 0°].

Figure 3 displays a detailed flow field in Region A2 at *z* = 2 m. As the axis of symmetry of the airflow field is the axis of *y* =135 m, Figure 3 only shows the streamlines in half of the zone. The urban design of open space decreases the wind speed and complicates the recirculation region (Figure 3c). On the contrary, the wind speed in Region A2 is strongly enhanced by the high-rise building (Figure 3e). The *VR* in Region A2 increases obviously compared with the base case, with a maximum value increase of 0.20. Both building layouts could deform the structure of the wind field around the building. Small vortices are formed near the windward side of the open space and the leeward side of the high-rise building.

**Figure 4:**
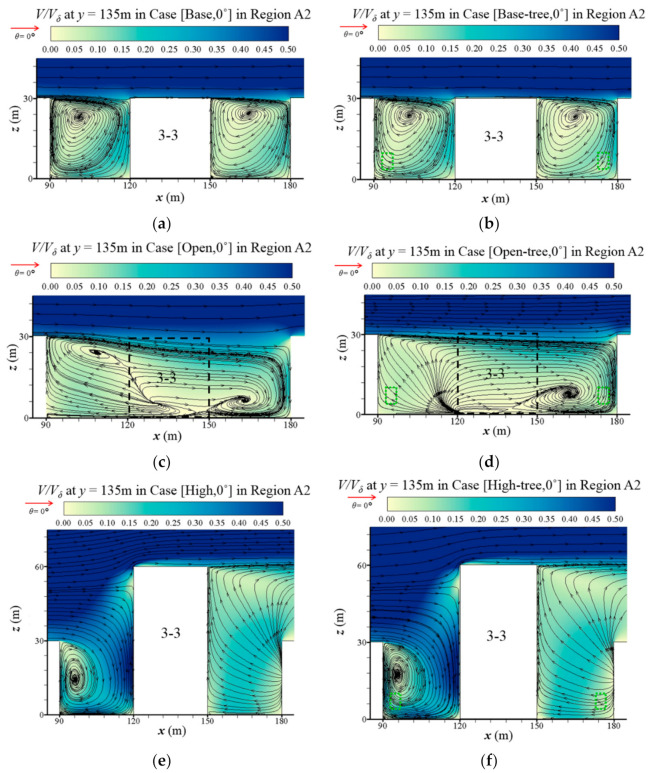
Normalised velocity (*V/V_δ_*, *V_δ_* = 4.34 m/s) at vertical plane (*y* = 135m) in Region A2 in (**a**) Case [Base, 0°], (**b**) Case [Base-tree, 0°], (**c**) Case [Open, 0°], (**d**) Case [Open-tree, 0°], (**e**) Case [High, 0°] and (**f**) Case [High-tree, 0°].

To determine the impact of building configurations on the airflow and the vortices’ structure, Figure 4a,c,e depicts the airflow and streamlines at the central *x*-*z* plane (*y* = 135 m) with the parallel approaching wind of Case [Base, 0°], Case [Open, 0°] and Case [High, 0°]. The designs of the open space and high-rise buildings have a significant influence on the geometry and the structures of the vortices in the street canyons, leading to obvious changes in the flow field. With the open space (Figure 4c), the vortex on the right side of Location 3-3 extends and occupies the space where Building 3-3 was. The vortex on the left side of Location 3-3 is compressed and the centre of the vortex rises up. Meanwhile, weakened airflows are observed compared with base case (Figure 4a). On the contrary, with the high-rise building (Figure 4e), the wind speed is significantly enhanced, especially near the windward side of the high-rise building. Compared with the base case, the former vortex on the left side of Building 3-3 is concentrated to one-half of the original size and the right vortex has disappeared.

#### 3.1.2. Influence of Tree Planting on Airflow

Figure 2b,d,f presents the flow field in the domain with tree planting. Whether for base cases (Figure 2a,b), open-space cases (Figure 2c,d) or high-rise building cases (Figure 2e,f), tree planting slightly changes the flow field in the entire domain (Region A1). Small vortices are generated over the whole domain, and the continuity of the flow is obstructed. However, the overall wind speed in Region A1 remains a similar value with the addition of vegetation under a parallel approaching wind.

This finding is further confirmed in Region A2, as presented in Figure 3. The wind vector around trees and street corners becomes denser and more complex. Tree planting slightly reduces the pedestrian-level wind speed in the central area of the base cases (Figure 3a,b) and open-space cases (Figure 3c,d). However, for high-rise building cases (Figure 3e,f), the VR value in Region A2 slightly increases with tree planting.

The vertical profiles of the flow field in Region A2 at y = 135 m are shown in Figure 4. We find that tree planting has a slight influence on the vertical airflow for all three building configurations. Compared with Case [Open, 0°], the two-vortices structure of the flow field is destroyed (Figure 4c,d). In the base cases (Figure 4a,b) and high-rise building cases (Figure 4e,f), the centre of the vortex on the left side of Building 3-3 rises up.

#### 3.1.3. Quantitative Analysis of Impact of Different Urban Layouts on Velocity Field

**Figure 5:**
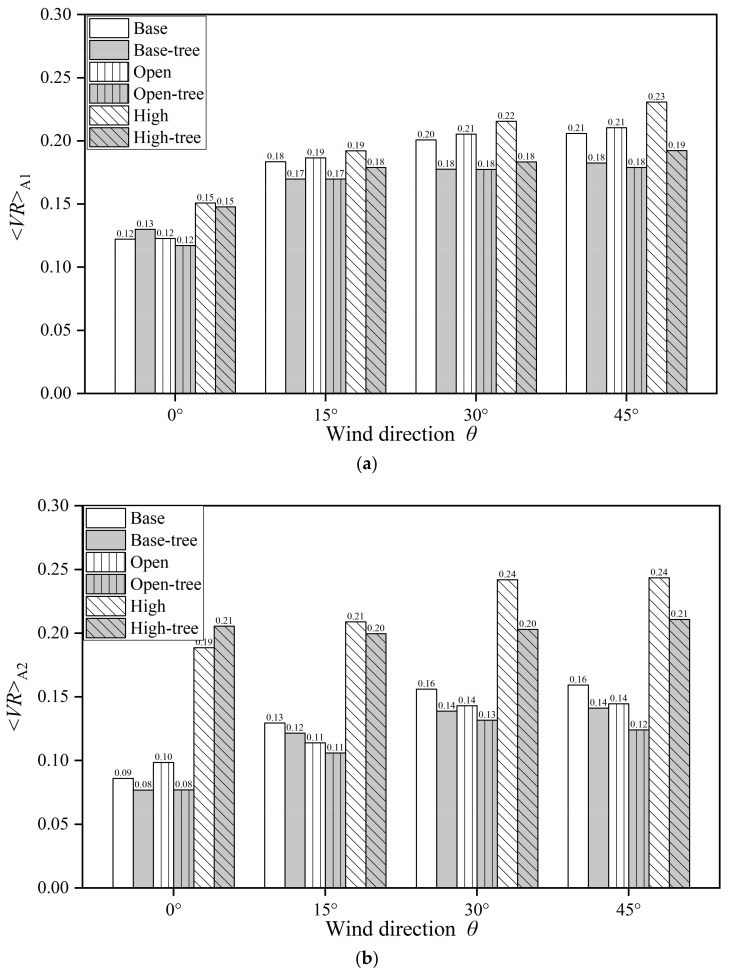
Spatially-averaged *VR* in different scenarios at *z* = 2 m in (**a**) Region A1 and (**b**) Region A2.

To quantify the impacts of different urban layouts on the flow field, Figure 5a,b and Table A5 summarise the spatial mean *VR* at *z* = 2 m of Region A1 (*<VR>*_A1_) and A2 (*<VR>*_A2_). Cases with different approaching wind directions (*θ* = 0°, 15°, 30° and 45°) are discussed as well. For Case [Base, *θ*], both 2 m *<VR>*_A1_ and *<VR>*_A2_ with the approaching wind direction of *θ* = 0° are much lower than those of other wind directions (*θ* = 15°, 30° or 45°). The maximum *<VR>*_A1_ (0.21) and *<VR>*_A2_ (0.16) both appear at *θ* = 45°.

For Case [Open, *θ*], the designed open space leads to a slight increase of 2 m *VR* on the spatial mean in Region A1 (Figure 5a) by 0.40–2.27%. However, in Region A2 (Figure 5b), the 2 m *VR* values in the open-space cases are decreased in comparison with those in base cases under the non-parallel approaching wind (*θ* = 15°, 30° and 45°), by 8.40–12.06%. Both the 2 m *<VR>*_A1_ and *<VR>*_A2_ of the open-space cases increase with the rising *θ*, with a maximum value of 0.21 and 0.14 at *θ* = 45°.

For Case [High, *θ*], the *VR* at *z* = 2 m has been significantly enhanced in both Region A1 and A2 compared with Case [Base, *θ*]. The ambient airflow of the high-rise building (*<VR>*_A2_) is enhanced by 52.78–119.05%, more strongly than that in the whole building matrix (*<VR>*_A1_) by 4.73–23.36%.

The results in Figure 5a,b and Table A5 show that tree planting reduces the *VR* in both Region A1 and A2 for different building configurations with all four approaching wind directions (*θ* = 0°, 15°, 30°, 45°), except Case [Base-tree, 0°] and Case [High-tree, 0°]. Tree planting significantly decreases the urban wind speed at *z* = 2 m (*<VR>*_A1_) on the basis of either open space or high-rise building designs, by 4.63–14.99% or 2.04–16.68%, respectively. The *<VR>*_A1_ with non-parallel approaching wind directions (*θ* = 15°, 30°, 45°) is reduced more in comparison with parallel approaching wind (*θ* = 0°). Taking Case [High-tree, *θ*] as an example, *<VR>*_A1_ is decreased by 6.87–16.68% compared with Case [High, *θ*] when *θ* ≠ 0°, more than the 2.04% when *θ* = 0°. These results are also consistent with the results presented in Figure 2, Figure 3, Figure 4 and Figure 5. Contrary to the reductive effect of most tree-planting cases, tree planting increases the *<VR>*_A1_ of Case [Base-tree, 0°] and *<VR>*_A2_ of Case [High-tree, 0°] by 6.27% and 8.98%, respectively. This phenomenon still needs more discussion in the ongoing work.

### 3.2. Impacts of Building Configurations and Tree Planting on Pollutant Dispersion

#### 3.2.1. Influence of Open Space and High-Rise Building on Pollutant Dispersion

**Figure 6:**
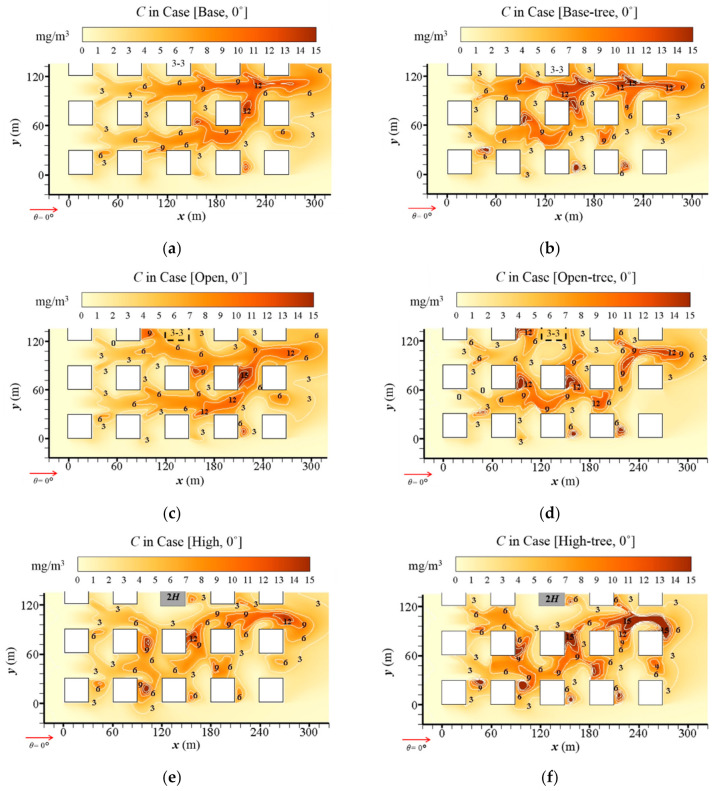
CO concentration (*C*) at *z* = 2 m in (**a**) Case [Base, 0°], (**b**) Case [Base-tree, 0°], (**c**) Case [Open, 0°], (**d**) Case [Open-tree, 0°], (**e**) Case [High, 0°] and (**f**) Case [High-tree, 0°].

To investigate the impacts of building configurations on pollutant diffusion, Figure 6a,c,e presents the distributions of CO concentration (*C*) at the pedestrian level (*z* = 2 m) in tree-free cases. Overall, the level of *C* in the three tree-free cases is similar, but the regions of high *C* are affected by different building configurations. In general, open space enhances the CO accumulation on the leeward side of the buildings in the central and downstream areas. Nevertheless, the high-rise building enhances the CO accumulation in the central and upstream area significantly. However, CO in the central area is diluted to a quite low level due to the strongly strengthened wind velocity surrounding the high-rise building.

**Figure 7:**
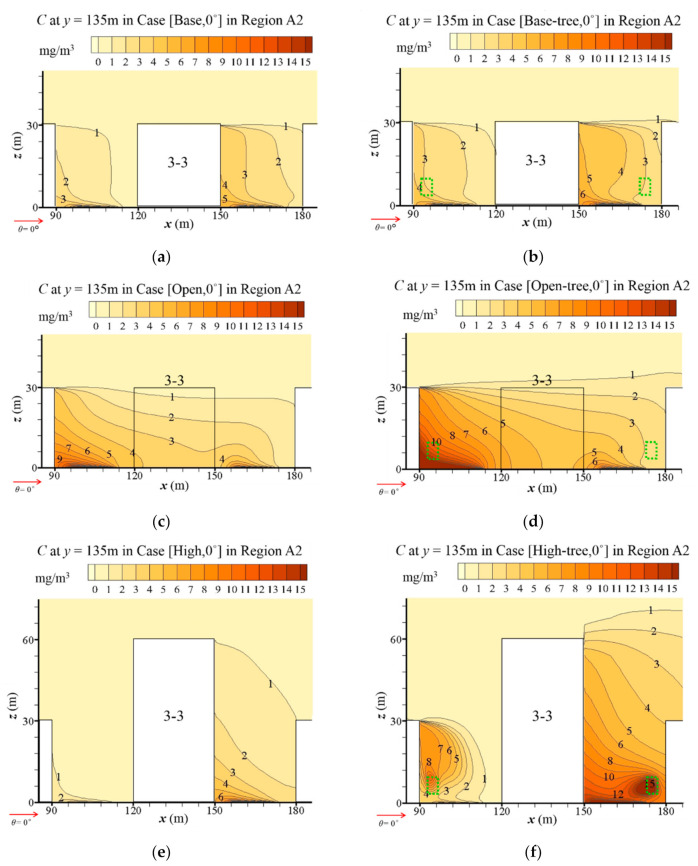
Vertical profile of *C* in Region A2 at *y* = 135 m: (**a**) Case [Base, 0°], (**b**) Case [Base-tree, 0°], (**c**) Case [Open, 0°], (**d**) Case [Open-tree, 0°], (**e**) Case [High, 0°] and (**f**) Case [High-tree, 0°].

To better understand how building configurations affect the pollutant dispersion in the central region (Region A2), Figure 7 shows the detailed vertical distribution of *C* at the *x-z* plane (*y* = 135 m). Comparing Figure 7c with Figure 7a, the low wind speed weakens the dilution and leads to high *C* levels in the open area. Particularly at the near-ground level of the upwind area, *C* is higher than 13 mg/m^3^, while *C* in the same area of the base case is about 5 mg/m^3^. In contrast, the strong airflow in the upwind of the high-rise building evidently decreases the *C* (Figure 7e). The near-ground *C* is decreased to about 2 mg/m^3^. Additionally, the CO distribution in the upwind of the high-rise building is reduced to a very limited vertical range, while that in the downwind is expanded due to the existence of the high-rise building.

#### 3.2.2. Influence of Tree Planting on Pollutant Dispersion

Figure 6b,d,f illustrates the *C* distribution in the urban area with tree planting, coupled with the basic design, open-space and high-rise-building design, respectively. Comparing them with Figure 6a,c,e, a significant increase of *C* is found in the whole urban area under the tree-planting design, no matter which type of building configuration is considered. The CO dispersion is significantly weakened by trees in the whole domain, and new hotspots with high *C* appear.

Detailed vertical distributions of *C* surrounding Building 3-3 in the central region (Region A2) with different building configurations are illustrated in Figure 7b,d,f. Compared with Figure 7a,c,e, tree planting evidently increases the near-ground *C* on the leeward side of all buildings. Particularly in Case [Open-tree, 0°], the near-ground *C* in the upwind of the open space increases to higher than 15 mg/m^3^. Trees around the high-rise building also have a significant influence on CO dispersion (Figure 7f). An area with a high *C* appears at the upper layer of the building wall on the upwind of Building 3-3, corresponding to the vortex of the flow field in Figure 4. At the downwind of Building 3-3, the ground-level *C* is higher than 15 mg/m^3^. Moreover, another hotspot with *C* higher than 15 mg/m^3^ appears in the area of the tree crown.

#### 3.2.3. Quantitative Analysis for Impact of Building Configurations and Tree Planting on Pollutant Dispersion

**Figure 8:**
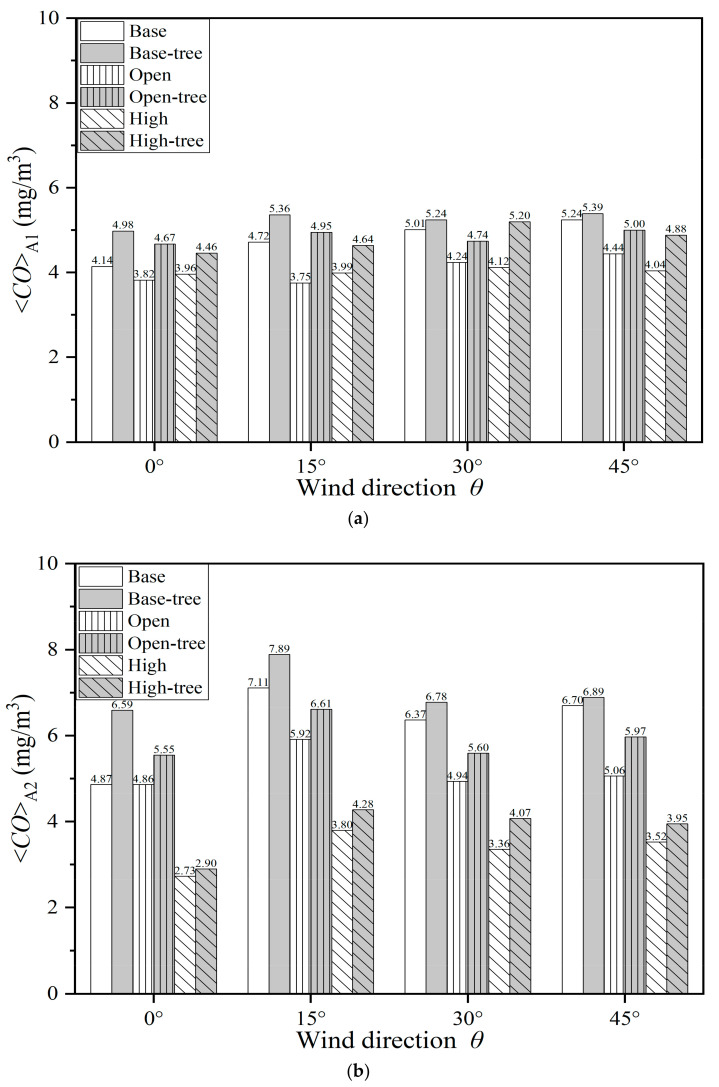
Spatially-averaged CO concentration in different scenarios at *z* = 2 m in (**a**) Region A1 and (**b**) Region A2.

Figure 8a,b and Table A6 summarise the mean CO concentration (*C*) at the pedestrian level (*z* = 2 m) in Region A1 (<*CO*>_A1_) and A2 (<*CO*>_A2_). The impacts of three building configurations, tree planting and wind directions (*θ* = 0°, 15°, 30° and 45°) are quantitatively assessed.

For the base cases, <*CO*>_A1_ slightly rises from 4.14 mg/m^3^ to 5.24 mg/m^3^ with the *θ* varying from 0° to 45°. <*CO*>_A2_, with the range of 4.87–7.11 mg/m^3^, is higher than <*CO*>_A1_ with the same direction of approaching flows. Both <*CO*>_A1_ and <*CO*>_A2_ decrease in the cases with open space and high-rise buildings (Figure 8a,b). For the open-space cases, <*CO*>_A1_ and <*CO*>_A2_ decrease rapidly with all four wind directions compared with the base cases, by 7.83–20.54% and 0.08–24.43%, respectively. For the high-rise building cases, the decrement ranges from 4.39% to 23.00% for <*CO*>_A1_, and ranges from 43.88% to 47.40% for <*CO*>_A2_, in comparison with base cases. The wind direction influences *C* more significantly for the area around the high-rise building (Region A2) than for the whole domain (Region A1).

For cases with tree planting, the results show that both the <*CO*>_A1_ and <*CO*>_A2_ evidently increase in all conditions with increasing rates of 2.84–31.88% and 2.85–35.46%, respectively. Taking *θ* = 0° as an example, <*CO*>_A1_ increases by 20.19%, 22.44% and 12.61% in Case [Base-tree, 0°], Case [Open-tree, 0°] and Case [High-tree, 0°], compared with the tree-free cases. For the base cases, the largest increasing ratios of <*CO*>_A1_ and <*CO*>_A2_ both appear when *θ* = 0°. With tree planting, the CO concentration at the pedestrian level and in the central area (<*CO*>_A2_) are higher than in the entire urban area (<*CO*>_A1_) for both the base cases and open-space cases. The <*CO*>_A2_ is particularly high in open-space cases with tree planting, with the values ranging from 6.59 mg/m^3^ to 7.89 mg/m^3^. For the high-building cases with tree planting, both <*CO*>_A1_ and <*CO*>_A2_ increase by 12.61–26.38% and 6.10–21.19%, respectively, compared with the tree-free cases. Regardless of the building configuration, the tree-planting design obviously weakens the dilution and dispersion capacity of pollutants and remarkably increases the CO concentration.

### 3.3. Impacts of Building Configurations and Tree Planting on <P_IF>

As illustrated in Section 2.1.2, we use <*P_IF*> to quantify the influence of urban layouts on personal exposure in UCL. As the buildings are assumed to be a residential type with natural ventilation, the pollutant concentration (*C*) on the building surfaces is adopted as the indoor concentration due to I/O ≈ 1 [6,7].

**Figure 9:**
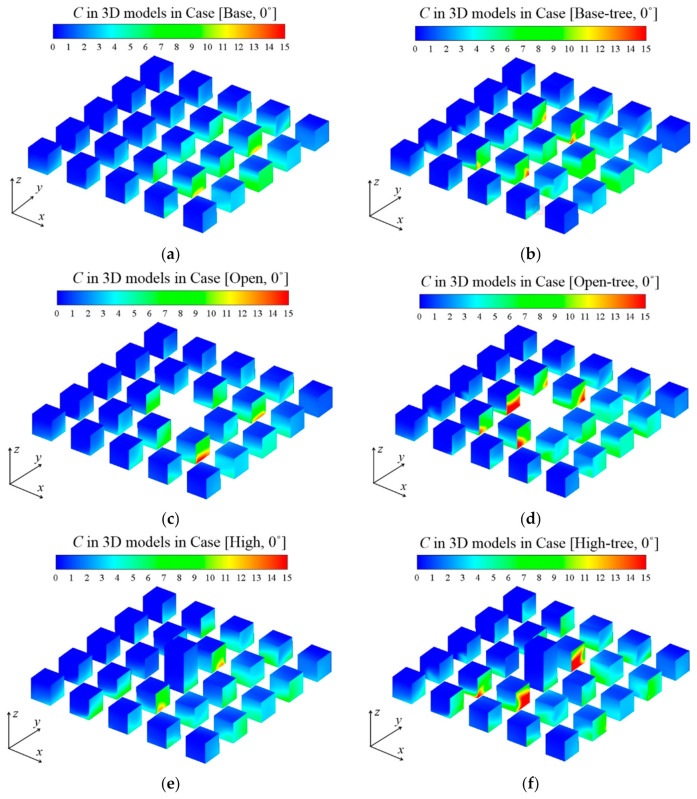
CO concentration (*C*) at building walls in 3D models: (**a**) Case [Base, 0°], (**b**) Case [Base-tree, 0°], (**c**) Case [Open, 0°], (**d**) Case [Open-tree, 0°], (**e**) Case [High, 0°] and (**f**) Case [High-tree, 0°].

**Figure 10:**
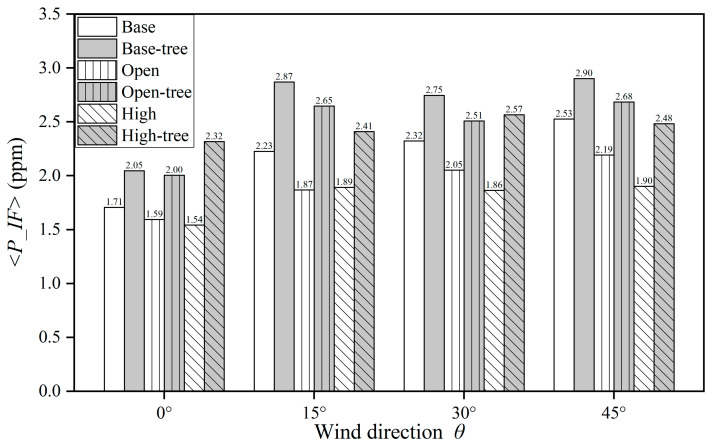
Building intake fraction <*P_IF>* in all cases.

Figure 9a–f plots *C* on the building surfaces in different cases when *θ* = 0°. The *C* distribution is apparently influenced by different building configurations and tree planting, especially in the central area. Figure 10 and Table A7 summarise the parameter <*P_IF*> to specify and quantify the CO exposure under scenarios with base conditions, open space, high-rise building and tree planting. Four wind directions (*θ* = 0°, 15°, 30°, 45°) are also considered in the evaluation.

As displayed in Figure 6a,c,e, the open space and high-rise building change the *C* distribution on the building surfaces. Both layouts make CO accumulate in the centre of the building matrix, while regions of high *C* in the base cases are in the downstream area of the approaching flow (Figure 9a–f). Comparing Figure 6b,d,f with Figure 6a,c,e, tree planting significantly increases the *C* on building surfaces, especially of the central 3 × 3 building matrix.

Similar results can also be found in Figure 10 and Table A7. For tree-free cases, both open space and high-rise building could decrease *<P_IF>* with the wind from all four directions by 6.56–16.08% and 9.59–24.70%, respectively. For different wind directions, the maximum *<P_IF>* of the tree-free cases always appears when *θ* = 45°, with *<P_IF> =* 2.53 ppm ([Base, 45°]), 2.19 ppm ([Open, 45°]) and 1.90 ppm ([High, 45°]), respectively. For cases with tree planting, the personal exposure in all building configurations (~2.05–2.90 ppm) is significantly increased compared with those of the tree-free cases (~1.54–2.53 ppm). The increasing ratio of *<P_IF*> ranges from 14.89% to 50.19% compared with tree-free cases. The maximum *<P_IF>* among all cases is 2.90 ppm, appearing in Case [Base-tree, 45°], while the maximum increasing ratio of *<P_IF>* is 50.19%, in Case [High-tree, 15°].

### 3.4. Velocity, CO Concentration and <P_IF> in Surrounding Area of A2 (Region A1−A2)

Table A8 and Table A9 summarise *VR* and CO concentrations in the surrounding area of A2 (Region A1−A2) in terms of spatial mean. In most of the scenarios, the building configurations and tree-planting plans have similar impacts as those in Region A2. However, the opposite effects exist in Region A1−A2 with certain conditions, especially for Case [Open-tree]. The *VR* values are increased in this region with all four wind directions, while the *VR* values in Region A2 are restrained for Case [Open-tree]. Meanwhile, the CO concentration of Case [Open-tree] in Region A1−A2 is decreased accordingly. The changes of the flow and dispersion are probably induced by the channelling effect owing to the narrowed street between the boundary and the open space. The phenomenon still needs more discussion in the ongoing work. Furthermore, the CO concentration in Region A1−A2 of Case [Open] is increased contrarily to that of Region A2, although most of the *VR* in Region A2 of Case [Open] is decreased. However, the open space improves the dilution conditions in Region A2. Thus, open space has the opposite impact on CO dispersion in the central area (Region A2) compared with the surrounding area (Region A1−A2). Moreover, since the variation of <*P_IF*> is closely related to CO concentration, the <*P_IF*> of Case [Open] will increase and that of Case [Open-tree] will decrease in Region A1−A2. It is also the opposite of that in Region A2.

## 4. Discussion

As critical determinants for urban ventilation and pollutant dispersion, the impacts of tree-planting plans and varied aspect ratios of 2D street canyons have been investigated in previous studies through both field experiments [93] and numerical simulations [80]. Chen et al. investigated the effect of different tree-planting parameters on the urban thermal and wind environment by conducting scaled outdoor field experiments [93]. Tree planting was found to reduce the pedestrian-level wind velocity in street canyons with all investigated *AR* values. The decreasing rate ranged 29–70%. Although the experiments are conducted in 2D idealised street canyon models, we also find that tree planting has a restraining effect on the urban wind in our work as well. Yang et al. [80] evaluated the integrated impact of tree planting and various *AR* values in a full-scale street canyon by CFD modelling (standard *k-ε* model) with the same emission settings as ours, and concluded that tree planting can lead to the reduction of velocity by various magnitude and an increase in CO exposure. In the canyon with *AR* = 1, the tree-induced CO increment is almost 70% (from 9.63 mg/m^3^ to 16.30 mg/m^3^). However, in 2D street canyon models, only the condition with perpendicular approaching wind to the street axis is considered, which corresponds to the worst ventilation situation, since only air exchange across the street roof contributes to pollutant removal. In this work with a 3D urban canopy, the ventilation can be better than in 2D models and is closer to that of the real urban community. With our 3D UCL model, the largest decreasing rate contributed by the tree planting is about 22% in spatial mean (Case [Open-tree, 0°] vs Case [Open, 0°]). Meanwhile, the tree-induced CO increment ranges 2.84–35.46% in this work (Table A6). We can conclude that even with the same *AR* (*AR* = 1) and tree-planting plan (tree planting on both sides of the street), the natural ventilation and dispersion conditions in the 3D building matrix are much better than that in 2D street canyons.

Using dimensional variables to evaluate the variance of the residents’ exposure owing to the varied impactors in different cities has huge challenges, because the emission strengths of different sources are not the same and may even be at different orders. Meanwhile, the size of the target population in different studies may vary significantly. As mentioned in Section 2.1.2, the variable *IF* has been used to express the source-to-intake relationship for vehicular pollutants in realistic street canyons [48], but this would be strongly affected by the population size and the spatial scale. For example, Habilomatis and Chaloulakou [45] found that the *IF* of vehicular ultrafine particles is 371 ppm in a 2D street canyon in the central area of Athens. The *IF* at the city scale is relatively small. Marshall et al. [94] reported that the *IF* of particles in US cities ranges from 1 to 10 ppm and the *IF* of CO is 270 ppm in Hong Kong, with a huge population size [46]. The *IF* of particles at the regional scale are reported to range from 0.12 to 25 ppm in the entire United States [95].

Consequently, the variable *<P_IF>* is derived and applied for the exposure assessment in this work. This normalised exposure index <*P_IF*> is more suitable for evaluating and comparing the exposure risks in different areas, since the influence caused by different orders of the population size and pollutant emission rates is avoided. Hang et al. found the *<P_IF>* of CO in the tree-free idealised 2D street canyon (*AR* = 1) was 5.21 ppm [42]. Yang et al. found the *<P_IF>* of CO in tree-planted 2D street canyons (*AR* = 1, *LAD* = 1) were 5.60 and 5.58 ppm, and the values raised with increased *AR*. When *AR* is raised to 5, the <*P_IF*> is an order of magnitude larger than that with *AR* = 0.5, 1 and 3. Comparing these works in 2D street canyons, the *<P_IF>* of CO in the 3D UCL model ranges from 1.54 to 2.87 with various tree-planting plans, building configurations and wind directions. The values in all scenarios are much lower than those in 2D street canyons.

According to the comparison above, an important suggestion for the urban designer is to avoid building 2D street canyons either too deep or too long in urban districts. If it cannot be avoided, more leakages and a wider roadway could improve the ventilation conditions in 2D street canyons. Moreover, if the street canyon is designed longer than 8H [17], the axis of the street should be approximately parallel to the prevailing wind direction.

To simplify the calculation process, we adopt idealised 3D UCL models in this paper. The building models are all assumed to be residential-type and are highly simplified with the same configuration (in a 5 × 5 building array) for the case study. The trees are treated as cubes of porous media. Neutral atmospheric conditions are adopted, and inert gas (CO) is considered as the tracer pollutant from the traffic emissions. Only four wind directions (*θ* = 0°, 15°, 30°, 45°) are considered in this research. Nevertheless, the real urban environment is affected by various parameters. Thus, it is worth mentioning that the results may be significantly different if urban morphologies, atmospheric conditions or other parameters are changed.

The impacts of urban morphological parameters in realistic urban areas are much more complicated than in such an idealised model. The study of different building coating plans and the direct radiation effect of the aerosol within urban canopy, as well as their impacts on the urban thermal environment and human outdoor thermal comfort (Figure 3 and Figure 4), is being implemented now. In future work, more kinds of realistic factors and conditions will be carefully considered and evaluated, including non-neutral atmospheric conditions and radiation impacts, the chemical reactions and composition of air pollutants, and more complicated urban morphological arrangements. Furthermore, the different tree species and the pollutant deposition on trees will also be considered in the ongoing work. CFD simulations coupling turbulence and radiation models will be validated by our scaled outdoor experiments (*H* = 1.2 m), as reported by Chen et al. [96,97]. These works will be adopted in numerical studies for full-scale realistic or idealised urban models. Our work is a step-by-step approximation of the real urban situation using the idealised model, and we are constantly improving our work on the way towards approaching the final target.

## 5. Conclusions

This paper is novel in that it numerically investigates the integrated impacts of open space, high-rise buildings and tree planting on urban airflow, pollutant dispersion and related human exposure in 3D idealised UCL models (5-row and 5-column, aspect ratio *H*/*W* = 1, building plan area fraction *λ_p_* = frontal area aspect ratio *λ_f_* = 0.25) under neutral atmospheric conditions. Four approaching wind directions (parallel 0° and non-parallel 15°, 30°, 45°) are considered. The computational fluid dynamics (CFD) simulations with the standard *k-ε* model are well validated by the wind tunnel data from the literature. The personal intake fraction *P_IF* and its spatially-averaged value for the entire UCL building surfaces <*P_IF*> are adopted to quantify the pollutant exposure on residents.

The CFD simulation results show that open space, high-rise building and tree planting all have strong effects on the flow structure, pollutant dispersion and residents’ exposure. Some meaningful findings are concluded as follows:

(1) Without tree planting, in contrast to the general 5 × 5 uniform-height building cluster (*H* = *B* = *W* = 30m), open space (the central building is removed) increases the spatially-averaged velocity ratio (*VR*) for the whole urban area under all four approaching wind directions (0°, 15°, 30° and 45°) by 0.40–2.27%. Designing the central building to be taller (2*H*) than the surroundings (*H*) can increase the *VR* for the entire urban area by 4.73–23.36%. In particular, the mean wind speed at the pedestrian level (*z* = 2 m) in the area around the high-rise building is significantly increased by 52.78–119.05%. However, tree planting significantly decreases the urban wind speed at *z* = 2 m on the basis of either open space or high-rise building designs, by 4.63–14.99% or 2.04–16.68%, respectively.
(2) Pollutant dispersion is determined by urban airflow characteristics. CO is released near the ground as a surrogate of traffic emissions. Without tree planting, both open space and central high-rise building would decrease the mean *C* at the pedestrian level for the whole urban area by 7.83–20.54% (0.32–0.97 mg/m^3^) and 4.39–23.00% (0.18–1.2 mg/m^3^) separately. This decreasing effect on *C* is significantly stronger for the high-rise building in the central area, by 43.88–47.40% (2.14–3.18 mg/m^3^). On the contrary, urban tree planting evidently weakens the pollutant dilution in all scenarios, with the increasing rate of 2.84–31.88% (0.15–1.2 mg/m^3^) for *C* at the pedestrian level in the entire urban area.
(3) The traffic-related CO exposure on residents in kerbside buildings is evaluated by *<P_IF*>. For the tree-free scenarios, both open space and high-rise buildings could decrease *<P_IF>* with the wind from all four directions by 6.56–16.08% and 9.59–24.70%, respectively. In contrast, tree planting obviously increases personal exposure in all scenarios by 14.89–50.19%. The *<P_IF*> of the tree-free cases ranges from 1.54 to 2.53 ppm, while *<P_IF*> ranges from 2.05 to 2.90 ppm in cases with tree planting.

This work provides a practical and efficient method to investigate the impacts of synthetic urban layouts on urban ventilation and pollutant dispersion. This work also extends the application of the CFD methodology to the assessment of exposure, and consequently connects to the area of public health. The method is applicable for further study coupling with more kinds of urban configurations under various atmospheric conditions. The results can provide helpful references for urban designers developing the sustainability of the city.

## Figures and Tables

**Table 1:** Scenarios tested in this work. Different urban designing and wind direction are considered for the assessment.

Building Arrangement	Vegetation Planning	Wind Direction (*θ*)	Case Name
Base	Tree-free	0°, 15°, 30°, 45°	[Base, *θ*]
	With tree	0°, 15°, 30°, 45°	[Base-tree, *θ*]
Open space	Tree-free	0°, 15°, 30°, 45°	[Open, *θ*]
	With tree	0°, 15°, 30°, 45°	[Open-tree, *θ*]
High-rise building	Tree-free	0°, 15°, 30°, 45°	[High, *θ*]
	With tree	0°, 15°, 30°, 45°	[High-tree, *θ*]

## Nomenclature

*AR*, *H/W*	aspect ratio
*B*, *H*, *W*	building width, building height and street width (m)
*Br*	volume-mean breathing rate (m^3^/s)
*C*	time-averaged pollutant (CO) concentration (kg/m^3^)
*C_d_*	leaf drag coefficient
<*CO*>_A1,_ <*CO*>_A2_	the spatial mean CO concentration at z = 2 m in Region A1 and in Region A2 (mg/m^3^)
*D_m_*, *D_t_*	molecular and turbulent diffusivity of the pollutant (m^2^/s)
*I*/*O*	indoor/outdoor pollutant concentration ratio
*IF*	intake fraction
*k*, *ε*	turbulent kinetic energy (m^2^/s^2^) and its dissipation rate (m^2^/s^3^)
*LAD*	leaf area density (m^2^/m^3^)
*m*	total pollutant emission over the considered period (kg)
*M*, *N*	total number of micro-environment types and population age groups
*P*	number of the population
*P*_*IF*, <*P*_*IF*>	personal intake fraction (ppm), building intake fraction (ppm)
*Re*	Reynolds number
*S*	realistic CO emission rate (kg/m^3^/s)
*S_Ct_*	turbulent Schmidt number
$S_{\bar{u_{i}}}$, *S_k_*, *S_ε_*	additional source and sink terms of momentum, *k* and *ε* (kg/m/s^3^)
*t*	time (s)
${\bar{u}}_{j}$	time-averaged velocity component (m/s) on stream-wise ($\bar{u}$), span-wise ($\bar{v}$, lateral) and vertical ($\bar{w}$) directions, as j = 1, 2, 3
***U***	velocity magnitude (m/s)
*U_in_(z)*	velocity profiles used at CFD domain inlet (m/s)
*U_ref_*, *u**	reference velocity at building height and friction velocity (m/s)
*v*, *v_t_*	kinematic viscosity and kinetic eddy viscosity (m^2^/s)
*V_p_*, *V_δ_*	wind velocity at pedestrian-level and at the top of boundary layer (m/s)
*VR*	velocity ratio
<*VR*>_A1__,_ <*VR*>_A2_	the spatial mean velocity ratio at *z* = 2 m in Region A1 and in Region A2 (mg/m^3^)
*x_j_*	spatial coordinates (m) on stream-wise (*x*), span-wise (*y*) and vertical (*z*) directions, as *j* = 1, 2, 3
*β_d_*	portion of turbulent kinetic energy converted from mean kinetic energy under the influence of drag
*β_p_*	dimensionless coefficient of the Kolmogorov cascade
*θ*	wind direction (°)
*κ_v_*	von Kármán constant
*λ_p_*, *λ_f_*	building plan area density, frontal area density
*ρ*	air density (kg/m^3^)

## Appendix A

1. Factors for exposure assessment
  **Table A1:** Population composition and related factors for exposure assessment.
  Item	Juveniles	Adults	Elderly
  Percentage of total population	21.2%	63.3%	15.5%
  Breathing rate *Br* indoors at home (m^3^/day)	12.5	13.8	13.1
  Time spent indoors at home (percentage)	61.70%	59.50%	71.60%
2. Validation studies for the flow, dispersion and vegetation modelling
  2.1. CFD validation of flow modelling
    **Table A2:** Statistical analysis between wind tunnel data and CFD simulation results—flow validation.
    Variable (Position)	Grid Size	Turbulence Model	NMSE *	FB **	R ***
    Criteria			≤1.5	−0.3–0.3	→ 1.0
    Stream-wise velocity at P1	Fine grid	STD	0.054	0.084	0.990
    	Medium grid	STD	0.049	0.087	0.990
    		RNG	0.107	0.018	0.996
    		RKE	0.115	0.094	0.986
    	Coarse grid	STD	0.038	0.081	0.991
    Stream-wise velocity at P2	Fine grid	STD	0.007	0.002	0.999
    	Medium grid	STD	0.009	0.002	0.997
    		RNG	0.045	−0.090	0.994
    		RKE	0.016	−0.083	0.998
    	Coarse grid	STD	0.011	0.013	0.997
    Stream-wise velocity at P3	Fine grid	STD	0.012	−0.063	0.996
    	Medium grid	STD	0.005	−0.024	0.998
    		RNG	0.093	−0.179	0.996
    		RKE	0.022	−0.100	0.997
    	Coarse grid	STD	0.005	−0.013	0.997
    Stream-wise velocity at P4	Fine grid	STD	0.014	−0.057	0.999
    	Medium grid	STD	0.014	−0.034	1.000
    		RNG	0.053	−0.175	0.998
    		RKE	0.014	−0.102	0.999
    	Coarse grid	STD	0.015	−0.023	0.999
    Stream-wise velocity at P5	Fine grid	STD	0.019	−0.051	0.998
    	Medium grid	STD	0.021	−0.017	0.998
    		RNG	0.033	−0.130	0.998
    		RKE	0.016	−0.073	0.997
    	Coarse grid	STD	0.022	−0.009	0.997
    Stream-wise velocity at P6	Fine grid	STD	0.037	−0.104	0.995
    	Medium grid	STD	0.037	−0.058	0.997
    		RNG	0.039	−0.147	0.993
    		RKE	0.039	−0.106	0.993
    	Coarse grid	STD	0.035	−0.048	0.998
    TKE at P1	Fine grid	STD	0.137	0.177	0.820
    	Medium grid	STD	0.115	0.150	0.842
    		RNG	0.275	0.343	0.662
    		RKE	0.361	0.366	0.727
    	Coarse grid	STD	0.091	0.115	0.867
    TKE at P5	Fine grid	STD	0.610	0.615	0.076
    	Medium grid	STD	0.621	0.615	0.135
    		RNG	1.299	0.837	−0.105
    		RKE	1.096	0.802	−0.378
    	Coarse grid	STD	0.526	0.575	0.255
    * normalised mean square error$:\;NMSE=\frac{\bar{{\left(Obs-Sim\right)}^{2}}}{\bar{Obs}*Sim}$. ** fractional bias-$FB=\frac{\bar{Obs}-\bar{Sim}}{0.5\;*\left(Obs+\bar{Sim}\right)}$. *** correlation coefficient–$R=\frac{\sum \left(Obs-\bar{Obs}\right)\left(Sim-\bar{Sim}\right)}{\sqrt{\sum {\left(Obs-\bar{Obs}\right)}^{2}\sum {\left(Sim-\bar{Sim}\right)}^{2}}}$.
  2.2. CFD validation of pollutant dispersion without tree models
    **Table A3:** Statistical analysis between wind tunnel data and CFD simulation results—dispersion modelling.
    Variable (Position)	NMSE *	FB **	R ***
    Criteria	≤1.5	−0.3–0.3	→ 1.0
    Leeward wall	0.021	−0.012	0.997
    Windward wall	0.064	−0.223	0.855
    Central line	0.029	−0.13	0.998
    * normalised mean square error: $NMSE=\frac{\bar{{\left(Obs-Sim\right)}^{2}}}{\bar{Obs}*Sim}$. ** fractional bias-$FB=\frac{\bar{Obs}-\bar{Sim}}{0.5\;*\left(Obs+\bar{Sim}\right)}$. *** correlation coefficient–$R=\frac{\sum \left(Obs-\bar{Obs}\right)\left(Sim-\bar{Sim}\right)}{\sqrt{\sum {\left(Obs-\bar{Obs}\right)}^{2}\sum {\left(Sim-\bar{Sim}\right)}^{2}}}$.
  2.3. CFD validation of pollutant dispersion with tree models
    **Table A4:** Statistical analysis between wind tunnel data and CFD simulation results—vegetation modelling.
    Variable (Position)		NMSE *	FB **	R ***
    Criteria		≤1.5	−0.3–0.3	→ 1.0
    Leeward side	*y/H* = 0	0.159	0.011	0.918
    	*y/H* = 2	1.306	0.594	0.987
    	*y/H* = 4	0.108	0.128	0.974
    Windward side	*y/H* = 0	0.055	−0.233	0.630
    	*y/H* = 2	0.007	0.049	0.784
    	*y/H* = 4	0.221	−0.256	0.700
    * normalised mean square error: $NMSE=\frac{\bar{{\left(Obs-Sim\right)}^{2}}}{\bar{Obs}*Sim}$. ** fractional bias-$FB=\frac{\bar{Obs}-\bar{Sim}}{0.5\;*\left(Obs+\bar{Sim}\right)}$. *** correlation coefficient–$R=\frac{\sum \left(Obs-\bar{Obs}\right)\left(Sim-\bar{Sim}\right)}{\sqrt{\sum {\left(Obs-\bar{Obs}\right)}^{2}\sum {\left(Sim-\bar{Sim}\right)}^{2}}}$.
3. Quantitative investigations of the flow, pollutant concentration and traffic-related exposure
  **Table A5:** Spatial mean velocity ratio (*VR*) at *z* = 2 m in Region A1 (<*VR*>_A1_) and Region A2 (<*VR*>_A2_) of each scenario and the change rate.
  Case	<*VR*>_A1_				<*VR*>_A2_			
  	0°	15°	30°	45°	0°	15°	30°	45°
  Case [Base]	0.12	0.18	0.20	0.21	0.09	0.13	0.16	0.16
  Case [Open]	0.12	0.19	0.21	0.21	0.10	0.11	0.14	0.14
  	+0.40%	+1.70%	+2.27%	+2.22%	+14.53%	−12.06%	−8.40%	−9.29%
  Case [High]	0.15	0.19	0.22	0.23	0.19	0.21	0.24	0.24
  	+23.36%	+4.73%	+7.30%	+12.07%	+119.05%	+61.30%	+55.06%	+52.78%
  Case [Base-tree]	0.13	0.17	0.18	0.18	0.08	0.12	0.14	0.14
  	+6.27%	−7.51%	−11.59%	−11.41%	−10.76%	−6.22%	−11.08%	−11.43%
  Case [Open-tree]	0.12	0.17	0.18	0.18	0.08	0.11	0.13	0.12
  	−4.63%	−9.01%	−13.65%	−14.99%	−21.92%	−6.91%	−7.84%	−14.18%
  Case [High-tree]	0.15	0.18	0.18	0.19	0.21	0.20	0.20	0.21
  	−2.04%	−6.87%	−14.93%	−16.68%	+8.98%	−4.42%	−16.11%	−13.44%
  The percentage number in each cell denotes the change rate of each case. Case [Base] is the comparison reference for Case [Open], Case [High] and Case [Base-tree]. The change rate of Case [Open-tree] refers to Case [Open], and that of Case [High-tree] refers to Case [High].
  **Table A6:** Spatial mean CO concentration (*C*) at *z* = 2 m in Region A1 (<*CO*>_A1_) and Region A2 (<*CO*>_A2_) of each scenario and the change rate.
  Case	<*CO*>_A1_ (mg/m^3^)				<*CO*>_A2_ (mg/m^3^)			
  	0°	15°	30°	45°	0°	15°	30°	45°
  Case [Base]	4.14	4.72	5.01	5.24	4.87	7.11	6.37	6.70
  Case [Open]	3.82	3.75	4.24	4.44	4.86	5.92	4.94	5.06
  	−7.83%	−20.54%	−15.31%	−15.27%	−0.08%	−16.82%	−22.48%	−24.43%
  Case [High]	3.96	3.99	4.12	4.04	2.73	3.80	3.36	3.52
  	−4.39%	−15.47%	−17.78%	−23.00%	−43.88%	−46.58%	−47.24%	−47.40%
  Case [Base-tree]	4.98	5.36	5.24	5.39	6.59	7.89	6.78	6.89
  	+20.19%	+13.55%	+4.73%	+2.84%	+35.46%	+10.93%	+6.47%	+2.85%
  Case [Open-tree]	4.67	4.95	4.74	5.00	5.55	6.61	5.60	5.97
  	+22.44%	+31.88%	+11.80%	+12.64%	+14.09%	+11.76%	+13.35%	+17.93%
  Case [High-tree]	4.46	4.64	5.20	4.88	2.90	4.28	4.07	3.95
  	+12.61%	+16.31%	+26.38%	+20.88%	+6.10%	+12.57%	+21.19%	+12.04%
  The percentage data denote the change rate of each case in contrast to Case [Base]. The change rate of Case [Open-tree] refers to Case [Open], and that of Case [High-tree] refers to Case [High].
  **Table A7:** Building intake fraction <*P_IF*> and the change rate in different cases.
  Case	*<P_IF>* (ppm)			
  	0°	15°	30°	45°
  Case [Base]	1.71	2.23	2.32	2.53
  Case [Open]	1.59	1.87	2.05	2.19
  	−6.56%	−16.08%	−11.65%	−13.19%
  Case [High]	1.54	1.89	1.86	1.90
  	−9.59%	−15.00%	−19.74%	−24.70%
  Case [Base-tree]	2.05	2.87	2.75	2.90
  	+19.94%	+28.92%	+18.24%	+14.89%
  Case [Open-tree]	2.00	2.65	2.51	2.68
  	+25.67%	+41.62%	+22.22%	+22.43%
  Case [High-tree]	2.32	2.41	2.57	2.48
  	+50.19%	+27.28%	+37.63%	+30.51%
  The percentage number in each cell denotes the rate of change of each case. Case [Base] is the comparison reference for Case [Open], Case [High] and Case [Base-tree]. The change rate of Case [Open-tree] refers to Case [Open], and that of Case [High-tree] refers to Case [High].
  **Table A8:** Spatial mean velocity ratio (*VR*) at *z* = 2 m in the Region A1−A2 (<*VR*>_A1−A2_) of each scenario and the change rate.
  Case Name	<*VR*>_A1−A2_			
  	0°	15°	30°	45°
  Case [Base]	0.55	0.83	0.90	0.92
  Case [Open]	0.59	0.76	0.79	0.81
  	+7.71%	−7.61%	−11.64%	−11.41%
  Case [High]	0.55	0.85	0.93	0.95
  	−7.90%	+11.35%	+16.88%	+16.60%
  Case [Base-tree]	0.53	0.77	0.79	0.81
  	−2.94%	−9.16%	−14.14%	−15.06%
  Case [Open-tree]	0.63	0.83	0.92	0.99
  	+19.69%	+6.92%	+15.91%	+23.36%
  Case [High-tree]	0.61	0.77	0.79	0.82
  	−3.82%	−7.20%	−14.76%	−17.11%
  The percentage number in each cell denotes the rate of change of each case. Case [Base] is the comparison reference for Case [Open], Case [High] and Case [Base-tree]. The change rate of Case [Open-tree] refers to Case [Open], and that of Case [High-tree] refers to Case [High].
  **Table A9:** Spatial mean CO concentration (*C*) at *z* = 2 m in the Region A1−A2 (<*CO*>_A1−A2_) of each scenario and the change rate.
  Case Name	<*CO*>_A1−A2_			
  	0°	15°	30°	45°
  Case [Base]	4.05	4.42	4.84	5.06
  Case [Open]	4.78	5.04	5.05	5.20
  	+17.89%	+14.07%	+4.45%	+2.83%
  Case [High]	3.69	3.48	4.15	4.36
  	−22.81%	−31.00%	−17.79%	−16.13%
  Case [Base-tree]	4.56	4.74	4.63	4.88
  	+23.82%	+36.15%	+11.57%	+11.87%
  Case [Open-tree]	4.11	4.01	4.21	4.10
  	−9.88%	−15.29%	−9.13%	−16.01%
  Case [High-tree]	4.65	4.69	5.34	5.00
  	+13.15%	+16.75%	+26.89%	+21.83%
  The percentage number in each cell denotes the rate of change of each case. Case [Base] is the comparison reference for Case [Open], Case [High] and Case [Base-tree]. The change rate of Case [Open-tree] refers to Case [Open], and that of Case [High-tree] refers to Case [High].
